# Validation of a Digital Support App to Assess Inflammatory Disease Activity and Mental Health Patient-Reported Outcomes (PROs): A Pilot Investigation

**DOI:** 10.1155/2019/7618468

**Published:** 2019-02-17

**Authors:** Kelly E. Tow, Claudia Rogge, Thomas Lee, Peter Caputi, Simon R. Knowles

**Affiliations:** ^1^Faculty of Social Sciences, University of Wollongong, Wollongong 2522, Australia; ^2^Department of Gastroenterology, Wollongong Hospital, Wollongong 2500, Australia; ^3^Illawarra Health and Medical Research Institute, Wollongong 2522, Australia; ^4^Faculty of Health, Arts & Design, Swinburne University of Technology, Hawthorn 3122, Australia

## Abstract

**Background:**

Real-time collection of mental health and disease activity patient-reported outcomes (PROs) are essential to assist clinicians in delivering optimal holistic health care. The aim of this pilot study was to validate a digital support tool (DST) delivered via a tablet device in an outpatient setting to assess IBD activity and psychological distress.

**Methods:**

48 individuals (26 females; average age: 40.04) with IBD completed the DST and a paper-based survey in a hospital IBD outpatient setting. PROs for disease activity and psychological distress (Kessler K10) were compared to paper-based gold-standard measures of disease activity (Partial Mayo Index or Harvey Bradshaw Index completed by the clinician) and psychological distress (Depression Anxiety Stress Scale; completed by the patient). Patient feedback regarding usability and acceptance of the DST was also collected.

**Results:**

DST patient-derived disease activity scores were significantly correlated with clinician assessment of disease activity (*p* < 0.01). Patient DST-derived psychological well-being scores were also significantly correlated with the gold-standard measure of psychological distress (*p* < 0.05). Patients found the DST to be easy to use and identified a willingness to follow through with the recommendations provided by the DST in relation to their psychological distress scores.

**Conclusions:**

The pilot study demonstrates the value in collecting disease activity and psychological distress PROs via a DST in an outpatient setting. Disease activity and psychological distress PROs were found to correlate significantly with gold standard measures. The findings provide preliminary support for the value of embedding digital technology into clinical care to promote patient engagement and optimal holistic healthcare.

## 1. Introduction

Recently within the inflammatory bowel disease (IBD) literature, an emphasis has been on the development and validation of patient-reported outcomes (PROs) [1, 2]. PROs provide researchers and clinicians with a validated collection of assessment tools which can be utilised in research trials and in general patient settings. The goal of such PROs is to assess multiple aspects of health, including physical and psychosocial outcomes derived from the patient [1]. To date, several PROs have been investigated. For disease activity, Jairath et al. [3] developed a 2-item-based PRO for ulcerative colitis (UC), while Khanna et al. [4] proposed a 2- and 3-item-based PRO for Crohn's disease (CD). Other PROs explored include disability [5], work productivity [6], anxiety, depression, and quality of life [2, 7].

In terms of patient settings, outpatient services provide an ideal opportunity to collect PROs. As recommended by several researchers [1, 2, 7], PROs collected in outpatient settings can provide clinicians with current and comprehensive biopsychosocial assessments, in turn allowing for optimal care, not only relating to physiological functioning but also other essential areas such as psychological comorbidity. In a recent systematic review, Mikocka-Walus and colleagues identified that up to 66% of IBD patients experience comorbid anxiety and/or depression [8]. Mental health issues have also been identified to exacerbate IBD activity [9–11] and increase the risk of disease flare-ups [10, 12–14]. Given the strong interrelationship between IBD and psychological comorbidity, and the growing evidence base identifying the impact of mental health on IBD, multiple IBD peak bodies have recommended standards of IBD care that include the review of and access to mental health services [15, 16].

The collection of disease activity and mental health PROs in an outpatient setting could provide real-time information to clinicians to facilitate a holistic optimal care approach. Consequently, we sought to develop a digital support tool (DST) application (or “app”) which could be delivered via a tablet device in an outpatient setting to assess both disease activity and mental health PROs.

The IBD patient assessment program (IBDPaP) is an android-based DST app developed by the senior author (SK) as a brief, easy to use, and flexible platform to record patient details. The DST collects a variety of biopsychosocial markers of well-being, including disease activity and psychosocial functioning. IBD symptom activity questions were derived from the Crohn's Disease Activity Index (CDAI) [17] and the Mayo Index [18]. The widely used and validated Kessler 10-item psychological distress scale (K10) [19] was used as the DST psychological distress PRO. IBDPaP also assesses patient suicidality (“do you consider hurting yourself, others, or feel suicidal? Yes/no”), current engagement with mental health services (“are you currently seeing a mental health professional? Yes/no”), and willingness to seek help (“do you have an appointment with a mental health expert within the next 14 days or are you willing to contact your local doctor (GP) if your mental health symptoms worsen?”). After completing the IBDPaP, patients are provided with feedback about mental health status. Summary reports can then be either emailed in PDF format to the patient's gastroenterologist or printed via a cloud device.

The aim of this pilot study was to validate a DST app delivered via a tablet device to assess patient-reported outcomes (PROs) for IBD activity and psychological distress. It was hypothesised that tablet-derived self-reported (1) disease activity PRO for UC (SR-PRO2-UC) [3] would significantly correlate with clinician verified partial Mayo Score [18] and PRO2 (PRO2-UC); (2) disease activity PROs for CD (SR-PRO2-CD and SR-PRO3-CD) [4] would significantly correlate with clinician-verified Harvey Bradshaw Index [20] and 2- and 3-item-based PRO for CD (PRO2-CD and PRO3-CD); (3) psychological distress (K10) would correlate significantly with a validated paper-based distress score (Depression Anxiety Stress Scale; DASS); and (4) patient feedback regarding mental health recommendations and app-based usability and usefulness would be positive.

## 2. Materials and Methods

### 2.1. Patients

Eighty-one participants (47 female, mean age 38.28, SD = 14.76; 48 with Crohn's disease) from an IBD outpatient service completed a DST. From the initial 81 participants, 48 (26 female, mean age 40.04. SD = 15.38; 25 with Crohn's disease; average number of years diagnosed 9.62) also completed a paper-based survey. One patient had a diagnosis of indeterminate colitis and was excluded from the analysis.

### 2.2. Measures

#### 2.2.1. Paper-Based Measures

*(1) Modified Harvey-Bradshaw Index (MHBI)*. The Harvey-Bradshaw Index (HBI) [20] is an efficient way to assess disease activity in CD patients. Respondents score 5 items based on how they felt the previous day. Items include general well-being, abdominal pain, number of liquid or soft stools per day, abdominal mass, and additional manifestations. In the present study, however, the modified HBI (MHBI) was used due to its improved simplicity and functionality. The MHBI is identical to the HBI, except that the question on abdominal mass is omitted, removing the need for physical examination of the patient. The MHBI has also been utilised in other cross-sectional studies with CD patients [21, 22]. Higher scores on the MHBI indicate greater disease severity, with scores equal to or below 5 indicating clinical remission.

*(2) Patient-Reported Outcome for Crohn's Disease (PRO2-CD and PRO3-CD)*. The 2- and 3-item PRO for CD (PRO2-CD and PRO3-CD, respectively) was based on items taken from the MHBI as described by Khanna et al. [4]: loose bowel movements [number of liquid or very soft stools] (×2), abdominal pain [0 = none, 1 = mild, 2 = moderate, 3 = severe] (×5), and for the third item, general well-being [0 = very well, 1 = slightly below par, 2 = poor, 3 = very poor, 4 = terrible] (×7). Higher scores indicated greater disease activity. For the 2-item PRO CD, remission was defined as a mean daily stool frequency ≤ 1.5 and abdominal pain score ≤ 1. For the 3-item PRO CD, the additional criterion for remission was a general well-being score of ≤1.

*(3) Partial Mayo Index (PMI)*. The Mayo Index [18] is a popular measure of ulcerative colitis disease activity. It includes stool frequency, rectal bleeding, mucosal appearance, and physician rating of disease activity. However, research suggests that the inclusion of endoscopic components, such as mucosal appearance, does not significantly contribute to the predictive value of the index [23]. Thus, in the current study, the noninvasive Partial Mayo Index (PMI) was used. All items are scored from 0 to 3, with the total being out of 9; scores equal to or below 1 indicate remission.

*(4) Patient-Reported Outcome for Ulcerative Colitis (PRO2-UC)*. The PRO2-UC was based on two items from the PMI as described by Jairath et al. [3]: stool frequency (normal [+0], 1-2 stools/day more than normal [+1], 3-4 stools/day more than normal [+2], >4/day more than normal [+3]) and rectal bleeding (none [+0], visible blood with stool less than half the time [+1], visible blood with stool half of the time or more [+2], passing blood alone [+3]). Remission was defined as rectal bleeding = 0 and absolute stool frequency ≤ 2.

*(5) Short Form of the Depression Anxiety Stress Scales (DASS21)*. The DASS21 is a validated measure of psychological distress [24, 25]. The scale consists of 21 statements, which assess individual distress in the domains of depression, anxiety, and stress. Respondents rate how much each statement applied to them over the past week, using a 4-point Likert scale, ranging from zero (did not apply to me at all) to three (applied to me very much or most of the time). A total distress scale score (0–126) is derived by summing the items and multiplying by 2 [26]. Higher scores indicate greater distress in the corresponding domains.

#### 2.2.2. DST-Based Measures

*(1) Disease Activity—Patient-Reported Outcomes (PROs)*. Similar to the paper-based UC PROs, the items for the SR-PRO2-UC were primarily based on the PMI. However, slight modifications based on gastroenterologist feedback were made to ensure patients were able to answer the questions most accurately and objectively. Based on these modifications, the 2-item disease activity score [3] was created (SR-PRO2-UC; how many bowel movements do you have per day? (0-2 stools/day [+0], 3 stools/day [+1], 4 stools/day more than normal [+2], >5 or more/day more than normal [+3]) and rectal bleeding (rectal bleeding over the last week: none [0], streaks of blood [+1], obvious blood [+2], mostly blood [+3]), with higher scores indicating greater disease activity. Remission was defined as rectal bleeding = 0 and absolute stool frequency ≤ 2.

**Figure 1:**
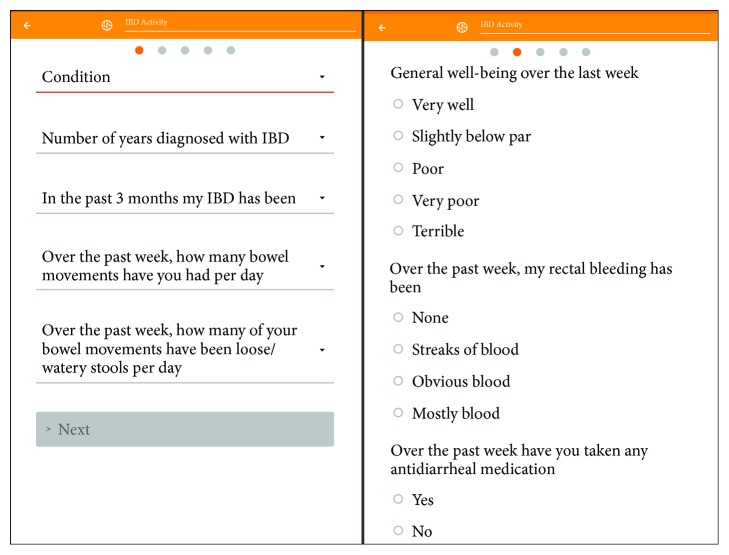
Example of IBD symptom activity questions presented in the IBDPaP and used in the calculation of disease activity PROs.

For CD patients, disease activity PRO scores [4] used HBI but modified items based on gastroenterologist feedback. The 2- and 3-item disease activity PRO scores (SR-PRO2-CD and SR-PRO3-CD) assessed bowel movements (how many of your bowel movements are loose/watery stools per day? [number of liquid or very soft stools] (×2)), abdominal pain/cramping (have you experienced abdominal pain or cramping over the last week? [0 = none, 1 = mild, 2 = moderate, 3 = severe] (×5)), and for the third item, general well-being over the last week [0 = very well, 1 = slightly below par, 2 = poor, 3 = very poor, 4 = terrible] (×2), with higher scores indicating greater disease activity. For the 2-item PRO CD, remission was defined as a mean daily stool frequency ≤ 1.5 and abdominal pain score ≤ 1. For the 3-item PRO CD, the additional criterion for remission was a general well-being score of ≤1. See Figure 1 for example IBD symptom activity questions presented in the IBDPaP.

**Figure 2:**
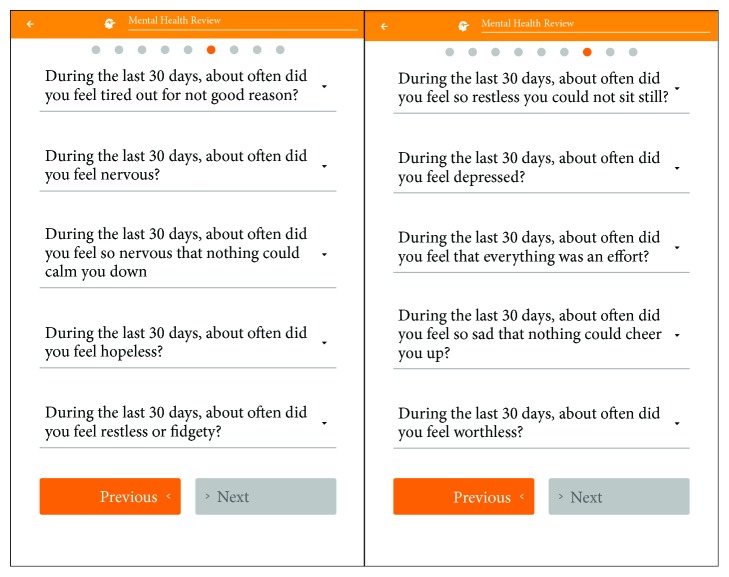
IBDPaP K10 screenshot.

*(2) Kessler Psychological Distress Scale (K10)–Patient-Reported Outcome (PRO)*. The Kessler Psychological Distress Scale (K10) [27] is a 10-item questionnaire, commonly used as an indicator of mental health status. Respondents answer a series of questions regarding their mental health experiences over the past 30 days, using a 5-point Likert scale from one (*none of the time*) to five (*all of the time*) (see Figure 2 for IBDPaP K10 screenshot). K10 scores between 10-15, 16-30, and 31-50 were identified with minimal, moderate, or severe psychological distress, respectively.

**Figure 3:**
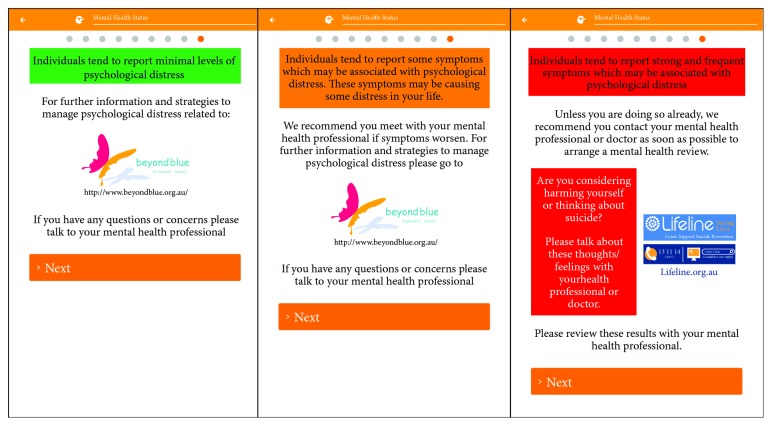
Mental health feedback responses, delivered dependent on the reported levels of psychological distress from low (left) and moderate (middle) to severe (right).

*(3) Suicidality*. A single question, “do you consider hurting yourself, others, or feel suicidal,” was also included into the tablet-based survey as a means to alert the clinician to any patients at heightened risk of self-harm or suicide. Patients who answered “yes” to this question would subsequently receive a feedback page which advised them to seek support and to talk with their doctor (see Figure 3). Additionally, patients' IBDPaP reports were emailed to their gastroenterologists, which ensured that appropriate referrals to mental health professionals could also be provided for patients deemed to be at increased risk.

*(4) Automatic Feedback and Recommendations Tablet-Based Psychological Distress Screening*. Based upon responses from the K10 and suicidality question, patients were presented with automatically generated feedback and recommendations (see Figure 3). If respondents identified any suicidality, they were provided with the same feedback provided to the severe K10 distress category.

*(5) DST Experience*. Eight questions assessed patient opinions and experiences in relation to using the DST (e.g., “how easy was it to use this tablet-based program?”) and receiving the automated feedback (e.g., “how helpful was this advice?” and “how likely are you to follow up on the advice that was provided?”), as well as the perceived benefit of integrating the DST into their ongoing care (e.g., “do you feel the information collected in this tablet-based program will help you and your gastroenterologist better manage your IBD in a collaborative way?” and “do you feel more motivated to manage your IBD after completing this tablet-based program?”). Questions were assessed on a 10-point Likert scale, e.g., 0 = ^“^not at all” to 10 = ^“^very much so.”

### 2.3. Procedure

Patients were approached at the IBD outpatient service. Consenting participants completed the IBDPaP DST on a Samsung 8” tablet and were then provided with a paper-based questionnaire. Patients could choose to complete the questionnaire while waiting for their appointment or complete at home within 24 hours and mail back using the prestamped and addressed envelopes. During the outpatient appointment, the clinician completed the patient's disease activity measures (i.e., PMI or HBI) in the paper-based questionnaire. Ethical approval was attained from the local university and hospital ethics committees prior to commencing the study.

### 2.4. Data Analyses

Analyses were conducted using IBM SPSS version 21. To allow for combined analysis of IBD disease activity across both CD and UC patients, the MHBI and PMI scores were first converted into Z-scores. Chi-square and *t*-tests were used to examine potential differences in demographic and psychometric variables between those who did and did not complete the paper-based questionnaire. Correlation analyses were performed to determine whether the tablet-derived disease activity PROs were associated with clinician-verified indices and paper-based PROs. Additionally, the correlation analysis examined whether the tablet-based psychological distress (K10) PRO was associated with the paper-based DASS21. Given that the assumption of normality was violated for several of the variables, Spearman's rho was utilised for all correlational analyses. Descriptive statistics on patients' experiences with the tablet-based survey were also explored.

## 3. Results

There were no significant differences in age, gender, or disease type and severity between those who did and did not complete the paper-based surveys. Similarly, there were no significant differences in psychological distress between these two groups. Of the 47 CD and UC patients who completed both the tablet- and paper-based surveys, the K10 scores identified 15 responders (31.9%) to be experiencing minimal psychological distress, while 27 (57.4%) had moderate psychological distress and 5 (10.6%) indicated severe psychological distress. Five participants (10.4%) identified positively to suicidal ideation and either not having an appointment with a mental health expert within the next 14 days or willing to contact their local doctor (GP) if their mental health symptoms worsen. All 5 participants were subsequently assessed during the gastroenterology appointment and provided an appropriate referral.

## 4. Relationships between Tablet- and Paper-Based Measures of Disease Activity and Psychological Distress

Prior to assessing relationships involving the disease activity PROs, differences in item format between gastroenterologist and patient self-completed disease activity items were evaluated. A series of *t*-tests indicated that there were no significant differences, suggesting similar responses by both gastroenterologists and patients despite the slight wording differences in items and response format.

There was consistency across each measure of disease activity for both CD and UC, with all related disease activity measures showing significant interrelations (see Table 1). Specifically, the tablet-derived PROs for CD demonstrated strong significant correlations with clinician-verified MHBI scores and paper-based CD PROs. Likewise, the tablet-derived PROs for UC were found to have a strong significant correlation with the clinician-verified Partial Mayo Index and the paper-based UC PRO although these correlations were stronger when using clinician-derived PRO scores (e.g. PRO2-CD) versus patient-derived PRO scores (e.g. PRO2-CDSR). Patient scores on the tablet-derived K10 showed a strong positive association with scores on the paper-based DASS21. This demonstrates that psychological distress scores were also consistent across tablet- and paper-based measures.

### 4.1. Accuracy of Remission Rates across Tablet- and Paper-Based Disease Activity Measures

Table 2 shows there was a strong overlap between the percentage of patients in remission across clinician-reported measures of disease activity and PROs for both CD and UC. While the PROs typically identified fewer patients as being in remission than the clinician-reported measures, the percentage overlap was similar between both the paper- and tablet-derived PROs.

### 4.2. Patient Perceptions of DST Experience

**Figure 4:**
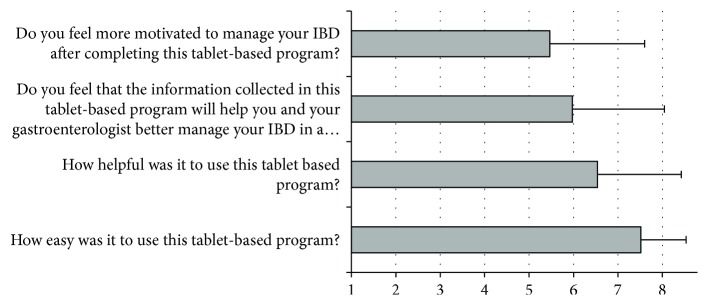
Patient perceptions on use of tablet-based program.

Examination of descriptive statistics on patients' experiences with the tablet-based survey (see Figure 4) suggested that most patients found the tablet easy to use (*M* = 7.51, *SD* = 1.02) and found it helpful (*M* = 6.54, *SD* = 1.89). Similarly, several patients felt motivated to manage their IBD after completing the survey (*M* = 5.46, *SD* = 2.13) and reported the information collected to be helpful to both themselves and their gastroenterologist in the management of their IBD (*M* = 5.98, *SD* = 2.07).

**Figure 5:**
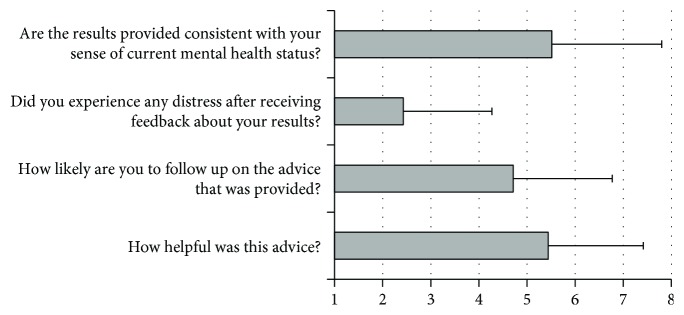
Patient perceptions on mental health feedback.

As shown in Figure 5, the results indicated that the instant feedback and advice were often perceived to be helpful (*M* = 5.44, *SD* = 1.99) and consistent with patients' sense of mental health (*M* = 5.51, *SD* = 2.28). While fewer patients reported that they would highly likely follow the advice given (*M* = 4.71, *SD* = 2.05), patients did not typically report high levels of distress after receiving the feedback (*M* = 2.43, *SD* = 1.85).

## 5. Discussion

Optimal management of a patient's well-being requires the collection of information in a timely and accurate way. DSTs are increasingly being identified as an important technology which can be embedded within traditional healthcare practice to help facilitate the monitoring and management of patient well-being. The current pilot study aimed to validate the IBDPaP, a DST designed to be utilised in IBD outpatient settings to (a) collect information regarding disease activity and mental health and (b) provide recommendations based upon participant mental health scores.

The results provided support for the first hypothesis that the IBDPaP DST patient-reported disease activity PROs for UC and CD significantly correlated with the clinician-derived disease activity PROs. Further, exploration of disease activity PROs dichotomised into either active or nonactive cases indicated that clinician-derived PROs could correctly identify remission status (compared against clinician-completed HMBI or PMI) in 72-80% of CD patients and 81.8% of UC patients. Likewise, consistency for patient-derived PROs was not much lower, with remission status identified correctly in 68% of CD patients and 77.3% of UC patients. Interestingly, this demonstrates that while clinician-derived PROs were understandably more accurate in classifying active or nonactive cases, the patient-reported PROs were correct in a high proportion of cases also. However, given that around 20% of cases are misclassified when using clinician-derived PROs, further work on developing these PROs is needed. Despite the identified limitations, the results provide preliminary support for the use of a DST to collect disease activity PROs. With further research, DSTs could become highly valuable and efficacious in informing clinicians about their patients' current IBD symptoms, severity, and perceived illness status. The findings also provide further evidence for the value of assessing PROs in outpatient settings [1, 2, 7].

Supporting the study's third and fourth hypotheses were the findings that the IBDPaP DST patient-reported psychological distress score was significantly correlated with the paper-based distress score (DASS21) and that patients perceived the IBDPaP DST as easy to use and helpful, whilst motivating them to better manage their symptoms. While patients identified that the feedback from the DST based upon their mental health scores was helpful, they reported being less likely to follow up on the advice provided. Importantly, of the participants who completed the IBDPaP DST, 5 participants (10.4%) identified positively to suicidal ideation and were subsequently assessed and referred appropriately. The findings suggest that while IBDPaP DST was accurate and helpful in collecting patient psychological distress status, its recommendations provided were not necessarily going to be followed up by patients. While these findings may reflect the reluctance of patients with IBD to seek mental health support [28, 29], it was clear that the collection of this data was helpful in identifying suicidality and informing clinicians about their patients' psychological well-being. The findings are also consistent with past research and recommended care standards identifying the importance of assessing mental health issues in IBD cohorts [8, 15, 16].

As identified above, an important finding from this pilot study was that patients respond positively to app-based usability and usefulness. Thus, while further research is important to determine the efficacy of the IBDPaP in improving features of patient care, these findings provide evidence that the integration of DST apps such as the IBDPaP can be well-accepted by patients and therefore hold great potential for improving patient and clinical management of IBD in the future.

However, the current study is not without limitations. While disease activity was assessed using validated measures, it did not utilise the full-scale and unmodified versions which include objective measures such as endoscopic information and biomarkers such as faecal calprotectin. Future research would benefit from utilising full-scale measures of disease activity along with objective measures and biomarkers. While it is essential that disease activity PROs are assessed, it is also worth noting that at this time, research validating such PROs is limited. Further, our study identified that the overlap between these PROs and standardised measures was low (e.g., 68% overlap for self-report CD PROs and MHBI). Clearly, further research is needed to develop well-validated and accurate disease activity PROs. In relation to patient completed PROs (PRO2CDSR, PRO3CDSR, and PRO2UCSR), due to the DST not differentiating between disease type, it instead asked the same disease-related questions. Consequently, to reduce patient question fatigue and improve patient understanding, a reduced number and combination of HBI and PMI items were used. Further research is needed to validate format and type of questions used to assess disease activity PROs as completed by patients using such tools as the DST. The study also did not assess the perceived utility of the IBDPaP DST as perceived by clinicians. Anecdotal feedback from clinicians was that the IBDPaP DST was easy to implement in the service, useful in helping to inform patient disease management plans, and provided a way to identify and explore patient perceptions relating to their physical and mental health. Future research should formally assess the perceptions of patients and clinicians in relation to the value of the IBDPaP DST in IBD outpatient settings. Future research could also explore the potential utility of adapting the DST to enable patients to provide disease activity (including faecal calprotectin values) and mental health symptoms on a regular basis at home along with data collection during an outpatient appointment. Finally, while the pilot study provides promising results, future research should be undertaken using a larger sample size stratified by disease class, gender, and age to evaluate the temporal reliability of the IBDPaP DST.

## 6. Conclusion

In the age of “apps” and patient empowerment, integrating DSTs to assess PROs prior to clinic visits will assist both patients and clinicians in focusing on what is important. Despite the small sample size, the study demonstrated strong correlations between IBDPaP DST patient-derived disease activity PROs and clinician-derived disease activity scores, as well as between patient-derived mental health PROs and a gold standard mental health measure. Further, the IBDPaP DST was able to alert clinicians to suicidal ideation in patients. An important finding from this pilot study was that patients found the IBDPaP DST to be beneficial, easy to use, and it was perceived that the app would assist patients and their gastroenterologists to better manage IBD in a collaborative way. Our study suggests that the IBDPaP is well received by patients and therefore presents a feasible approach for the facilitation of patient engagement and optimal holistic healthcare.

## Figures and Tables

**Table 1:** Spearman's rho correlations and descriptive statistics of disease activity and psychological variables.

	1	2	3	4	5	6	7	8	9	10	Mean (SD)
1. Standardized disease activity scores	—										0.04 (1.00)
2. MHBI (paper)	1.00^∗∗∗^	—									3.52 (3.29)
3. PRO2-CD	0.95^∗∗∗^	0.95^∗∗∗^	—								7.20 (6.17)
4. PRO3-CD	0.88^∗∗∗^	0.88^∗∗∗^	0.88^∗∗∗^	—							13.64 (12.20)
5. PRO2-CDSR (tablet)	0.81^∗∗∗^	0.81^∗∗∗^	0.82^∗∗∗^	0.78^∗∗∗^	—						8.92 (10.92)
6. PRO3-CDSR (tablet)	0.79^∗∗∗^	0.79^∗∗∗^	0.78^∗∗∗^	0.82^∗∗∗^	0.96^∗∗∗^	—					15.92 (17.26)
7. Partial Mayo (paper)	0.99^∗∗∗^	—	—	—	—	—	—				1.32 (2.08)
8. PRO2-UC	0.97^∗∗∗^	—	—	—	—	—	0.99^∗∗∗^	—			0.91 (1.11)
9. PRO2-UCSR (tablet)	0.56^∗∗^	—	—	—	—	—	0.55^∗∗^	0.58^∗∗^	—		1.50 (1.54)
10. K10 (tablet)	0.45^∗∗^	0.57^∗^	0.57^∗∗^	0.67^∗∗^	0.60^∗∗^	0.68^∗∗∗^	0.17	0.22	0.43^∗^	—	20.17 (8.21)
11. DASS21 (paper)	0.30^∗^	0.42^∗^	0.35	0.40	0.38	0.40^∗^	0.02	0.06	0.38	0.74^∗∗∗^	15.68 (13.79)

Note. ^∗^*p* < 0.05, ^∗∗^*p* < 0.01, ^∗∗∗^*p* < 0.001.

**Table 2:** Percentage of patients in remission across both CD and UC measures and PROs.

Crohn's disease				Ulcerative colitis			
	Sample (*n*)	Patients in remission (%)	Correct overlap with MHBI (%)		Sample (*n*)	Patients in remission (%)	Correct overlap with PMI (%)
MHBI	25	80.0%	—	PMI	22	72.7%	—
PRO2-CD	25	60.0%	80.0%	PRO2-UC	22	90.9%	81.8%
PRO3-CD	25	52.0%	72.0%	PRO2UCSR	22	59.1%	77.3%
PRO2CDSR	25	48.0%	68.0%				
PRO3CDSR	25	48.0%	68.0%				

## Data Availability

Deidentified data utilised in this study will be made available from the corresponding author upon request and after approval from local and university and hospital ethics committees.
